# Volatile Organic Compounds Produced by *Bacillus* sp. Strain R2 Inhibit *Aspergillus flavus* Growth In Vitro and in Unhulled Rice

**DOI:** 10.3390/foods13182898

**Published:** 2024-09-13

**Authors:** Qingyun Wang, Kaige Zhang, Lu Yu, Qinlu Lin, Wenhua Zhou

**Affiliations:** 1 College of Food Science and Engineering, Central South University of Forestry and Technology, Changsha 410004, China; kaigezhang604@csuft.edu.cn (K.Z.); 20201200555@csuft.edu.cn (L.Y.); t20081475@csuft.edu.cn (Q.L.); zwhcultue@126.com (W.Z.); 2 National Engineering Research Center of Rice and Byproduct Deep Processing, Changsha 410004, China

**Keywords:** *Bacillus paramycoides*, volatile organic compounds, unhulled rice, *Aspergillus flavus*, antifungal activity

## Abstract

Volatile organic compounds (VOCs) produced by *Bacillus* species exhibit biocontrol activity against fungal pathogens of fruits and vegetables. However, research on the effect of VOCs on *Aspergillus flavus* in stored grains is limited. This study aimed to investigate the effects of VOCs extracted from the strain R2, which was isolated from unhulled rice and identified as *Bacillus paramycoides* on *A. flavus* in vitro and unhulled rice. R2 VOCs effectively inhibited conidial germination and the hyphal growth of *A. flavus* in vitro. Moreover, R2 VOCs reduced the fungal population, aflatoxin B_1_ (AFB_1_) levels, and free fatty acid (FFA) value by 90.8%, 67%, and 38.7%, respectively, in unhulled rice. Eighteen R2 VOCs were identified using headspace solid-phase micro-extraction gas chromatography–mass spectrometry, and the individual activity of the VOCs against *A. flavus* was tested in vitro. Benzaldehyde (Ben) and 3,7-dimethyl-1-octanol (Dmo) showed strong inhibitory activities against *A. flavus* on PDA plates, with inhibition rates of 100% and 91.2%, respectively, at a concentration of 20 μL/dish. Ben at the concentration of 0.09 mg/mL, Dmo at the concentration of 0.07 mg/mL, or a mixture of both at halved concentrations could reduce the fungal population, AFB_1_ levels, and FFA content in unhulled rice. Our findings suggest that R2 VOCs are good alternatives to traditional chemical fumigants for suppressing *A. flavus* in stored grains. However, further research is necessary to establish the optimal fumigation concentration of these two components in unhulled rice. The impact of their residues on grain quality should be explored through sensory evaluation and nutritional analysis, and their safety to the environment and human body should be evaluated through safety assessment.

## 1. Introduction

Rice is a staple food for more than half of the world’s population, and unhulled rice is the main reserve grain in Southeast Asia. *Aspergillus flavus* and aflatoxin contamination seriously affect the yield and quality of rice grains [1,2,3]. *A. flavus* is one of the most frequently detected fungi in stored grains [4,5]. *A. flavus* can survive under dry conditions and dominate the fungal populations of stored grains owing to its xerophilic nature [6,7], thereby causing grain decay and aflatoxin accumulation under warm and humid conditions. Aflatoxins are a group of toxic secondary metabolites produced by a few fungi of the genus *Aspergillus* [8], mainly *A. flavus* and *A. parasiticus* [9]. Aflatoxin B_1_ (AFB_1_) is the most potent of all identified aflatoxins [10] and is classified as a group 1 carcinogen by the International Agency for Cancer Research [11] because of its potential carcinogenic [12], hepatotoxic [13], cytotoxic [14], and immunotoxic [15] properties.

Traditionally, physical and chemical methods have been used to control *A. flavus* and aflatoxin in stored grains [16,17]. Several fungicides, such as iprodione, benomyl, and thiabendazole [16,18], are used for fungal control during grain storage. However, the drug resistance of pathogenic fungi and the possible hazards of drug residues to consumers necessitate the need to explore alternative methods to reduce the use of traditional chemical products [19,20].

Most *Bacillus* species are environmentally friendly [21] and can produce a variety of antifungal substances, such as antifungal proteins [22], peptides [23,24,25], and volatile organic compounds (VOCs) [26]. Compared with antifungal proteins and peptides, VOCs are more suitable for the control of harmful fungi in stored grains, as they can volatilize at room temperature and normal pressure, thus easily entering grain piles and dispersing uniformly in stored grains. Additionally, VOCs are prone to volatilization and decomposition, resulting in low residual levels. However, research on *Bacillus* VOCs used for harmful fungi control in stored grains are currently limited. Previous studies have demonstrated the broad-spectrum antifungal activity of microbial VOCs. VOCs from *Bacillus megaterium* [2], *Pseudomonas protegens* [27], *Shewanella algae* [28], *B. megaterium* BM344-1 [29], *Aureobasidium* strains [30], and *Bacillus* strain TM-I-3 [31] effectively inhibit or kill *Aspergillus* spp. and minimize aflatoxin biosynthesis. These VOCs are also effective against other pathogenic fungi, such as *Penicillium fellutanum*, *Alternaria alternata*, and *Botrytis cinerea* [32]. These research results provide a theoretical basis for using VOCs from *Bacillus* species to control pathogenic fungi and related mycotoxins in stored grains.

Therefore, in order to explore safe and efficient microbial-derived antifungal VOCs and to investigate their effectiveness in controlling harmful fungi in stored grains, in this study, three *Bacillus* strains were isolated and strain R2 was selected for producing VOCs with the most potent antifungal activity against *A. flavus*. Furthermore, the antifungal effect of R2 VOCs against *A. flavus* in vitro and in unhulled rice was determined, the components of R2 VOCs were analyzed, and the identified individual compound was tested for its antifungal effects separately; benzaldehyde (Ben) and 3,7-dimethyl-1-octanol (Dmo) were selected for their potent antifungal activity against *A. flavus* in vitro. Finally, the inhibitory effect of Ben and Dmo on the fungal population, free fatty acid (FFA) value, and AFB_1_ content in unhulled rice was tested. To the best of our knowledge, this is the first report on the inhibitory effects of Ben and Dmo on *A. flavus* control in unhulled rice. The results will provide insights into developing safe and efficient solutions for harmful fungi control in stored grains.

## 2. Materials and Methods

### 2.1. Microorganisms and Culture Media

*Bacillus* strains were isolated from unhulled rice (strain R2), fermented sauerkraut (strain S2), and fermented bean curd (strain F1). Sterile water (50 mL) was added to a beaker containing 5 g of each material and was boiled for 3 min to kill spore-free microorganisms. Under sterile conditions, a 0.1 mL of the boiled liquid was spread on a beef extract-peptone agar (BPA) plate (containing 3 g beef extract, 10 g peptone, 5 g NaCl, and 20 g agar in 1 L distilled water, pH 7.3), and cultured at 37 °C for 48 h. Colonies of bacteria with spores were selected through smear tests and microscopic examination. The selected strains were screened for their ability to produce antifungal VOCs using a double Petri dish assay [33]. One plate contained BPA medium, and the other contained PDA medium. The selected strain was streaked onto a BPA plate, and a mycelial plug (6 mm diameter) from a 6-day-old culture of *A. flavus* was placed on the PDA plate. The plates were then sealed with polyethylene film. Plates inoculated with *A. flavus* alone served as the control. Three replications were used for each treatment. Plates were incubated at 30 °C for 5 days. The percentage of the colony radial growth inhibition of *A. flavus* colonies was measured, and the inhibition rate (%) was calculated using the formula:

[(dc − dt)/dc] × 100

where dt is the colony diameter of *A. flavus* in the treated plate and dc is the colony diameter of *A. flavus* in the control plate. Based on the screening results, strain R2 was found to produce antifungal VOCs. Next, the strain was subcultured on BPA medium at 30 °C for 24 h. Fresh cells of R2 were suspended in sterile water and adjusted to a final concentration of 10^7^ CFU/mL.

The pathogen *A. flavus* was obtained from the China General Microbiological Culture Collection Center (No. 3.6305). The fungus was transferred to a PDA slant and incubated at 30 °C for 7 days. Conidia were collected and suspended in sterile distilled water containing 0.03% (*v*/*v*) Tween 80, and the concentration of the suspension was adjusted to 10^5^ CFU/mL.

### 2.2. Identification of Strain R2

To identify strain R2, the following biochemical tests were performed: citrate utilization, catalase, Voges–Proskauer (VP), methyl red, indole, phenylalanine deaminase, mannitol fermentation, starch hydrolysis, gelatin liquefaction, and acid production from glucose and xylose [34]. Furthermore, the genomic DNA of R2 was extracted and 16S rDNA was amplified as previously described [35]. The PCR products were sequenced by Beijing Genomics Institution Tech in Wuhan, China. The 16S rDNA sequences were queried against the nucleotide sequence database of the National Center for Biotechnology Information (NCBI). Sequences with significant alignments in the NCBI BLAST result were selected, and MEGA7.0.26 was used to construct a neighbor joining (NJ) tree to determine the taxonomic status of strain R2.

### 2.3. Effect of VOCs Produced by R2 on Spore Germination of A. flavus

The effect of the VOCs produced by R2 on the conidial germination of *A. flavus* was examined using a double Petri dish assay [33], as described in Section 2.1. First, 0.1 mL of a 10^7^ CFU/mL R2 cell suspension was evenly spread on a BPA plate, and 0.1 mL of a 10^5^ CFU/mL conidia suspension of *A. flavus* was spread on a PDA plate. Sterile water (0.1 mL) instead of an R2 cell suspension was used as a control. Next, the two sealed plates were separately incubated at 30 °C for 8, 16, 48, and 72 h. The plates were observed using an inverted microscope (Leica DMi4000B, Wetzlar, Germany). Notably, 100 conidia were observed, and the number of germinated conidia was counted. The spore germination inhibition rate of the treated groups was calculated using the formula:

[(nc − nt)/nc] × 100

where nc is the number of conidia germinated in the 100 observed conidia in the control plate, and nt is the number of conidia germinated in the 100 conidia in the treatment plate.

### 2.4. In Vivo Test of A. flavus Decay Control by R2 VOCs

**Figure 1:**
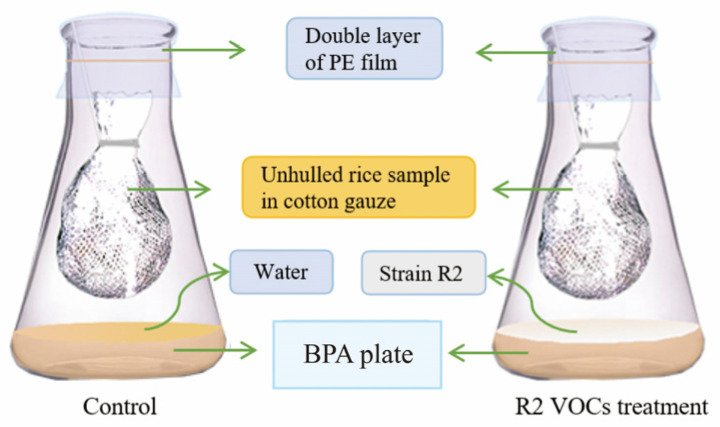
Diagram of the Erlenmeyer flask incubation method used to determine the inhibitory effect of R2 VOCs on *A. flavus* decay in unhulled rice samples.

We designed an Erlenmeyer flask incubation method to determine the inhibitory effect of R2 VOCs on *A. flavus* decay in unhulled rice samples (Figure 1). Briefly, 15 mL of BPA medium was added to a 100 mL Erlenmeyer flask, sterilized at 121 °C for 20 min, and allowed to cool to form a plate at the bottom. Next, 50 μL of a 10^7^ CFU/mL R2 cell suspension was smeared on the plate, and 50 μL sterile water was used as the control. Fresh unhulled rice samples were placed on a porcelain plate to form a thin layer and treated under UV light for 40 min to kill microorganisms on the sample. Then, 5 g of rice was inoculated with *A. flavus* conidia by spraying 500 μL of a 10^5^ CFU/mL *A. flavus* conidial suspension. The sprayed sample was wrapped in sterile cotton gauze and hung in a flask at a height of 2 cm from the flask bottom plate surface. The flasks were sealed with double layer polyethylene film and incubated at 30 °C for 3, 5, and 7 days, respectively. Experiments were performed in triplicate. The population of *A. flavus* and AFB_1_ and the FFA levels in the samples were determined.

*A. flavus* populations were examined as previously described [3]. Three replicates were performed, and the mean values are reported.

To examine the aflatoxin levels in the rice samples, they were finely ground in a mortar and passed through a 40 mesh sieve. Ground samples (10 g) were suspended in 20 mL of 70% methanol, followed by vigorous vortexing for 3 min. The suspensions were filtered, and an ELISA kit (Sangon Biotech Shanghai Co., Ltd., Shanghai, China) was used to determine AFB_1_ levels in the sample extracts following the manufacturer’s instructions. Each sample was measured thrice using a DNM-9602 microplate reader (Perlong Medical Equipment Co., Ltd., Beijing, China) at 450 nm. The AFB_1_ levels were calculated using the following formula:

(c × v)/m

where c is the AFB_1_ concentration in the sample extract (μg/L), v is the sample extract volume (L), and m is the dry weight of the sample (kg).

The FFA levels in the rice samples were determined using an acid-base titration method [36] with some modifications. FFAs were extracted using anhydrous ethanol, and the FFA levels were defined as the number of milligrams of KOH required to neutralize FFAs in 100 g of dry rice sample.

### 2.5. Analysis of the VOCs Produced by Strain R2

Headspace solid-phase micro-extraction gas chromatography–mass spectrometry (HS-SPME-GC-MS) was used to analyze the composition of VOCs produced by strain R2 as previously described [37]. Notably, 50 μL of 10^7^ CFU/mL R2 was spread on a modified NA medium slant in a 50 mL headspace bottle and cultured at 32 °C for 72 h. Headspace bottles containing uninoculated NA medium were used as controls. The bottles were transferred to a 40 °C water bath and equilibrated for 30 min. SPME fiber (50/30 μm DVB/CAR/PDMS, Supelco, Inc., Bellefonte, PA, USA) was preconditioned per the manufacturer’s instructions and exposed to the headspace of the bottle for 30 min at 40 °C. The trapped volatiles were analyzed using GC-MS (6890/5973N, Agilent Technologies Inc., Centerville Road, Wilmington, DE, USA) and a DB-5MS fused-C18 capillary column (Agilent Technologies Inc., Centerville Road, Wilmington, DE, USA).

The SPME fiber containing the trapped VOCs was immediately inserted into the injection port of the GC-MS system for thermal desorption at 250 °C for 2 min. VOC separation was performed using a capillary HP-5MS column (30 m × 0.25 mm × 0.25 μm). The GC settings were as follows: initial temperature, 33 °C for 3 min; heating to 180 °C at a rate of 10 °C/min; and heating to 240 °C at a rate of 40 °C/min for 4 min. Helium was used as the carrier gas; the running time was 23.2 min. The MS settings were as follows: the temperature of the ion source was 230 °C, the ion source was operated in electron impact mode at 70 eV, and the full scan mode was used for detection at the *m*/*z* range of 50–500. VOCs were identified via comparison with the National Institute of Standards and Technology mass spectral library, and the content of volatile compounds was quantified based on the peak intensity.

### 2.6. Analysis of the Activity of Identified Individual VOCs against A. flavus

The standard chemicals of the identified VOCs were purchased from Sigma-Aldrich and were individually tested against *A. flavus* using a double Petri dish assay as described in Section 2.1. Briefly, 5 μL of 10^5^ CFU/mL conidia suspension of *A. flavus* was inoculated in the center of the PDA plate, and 80 μL of each compound was pipetted into another plate. The two plates were immediately stacked, with the PDA plate on top and the other on the bottom. An empty plate was used as the control [37]. The plates were then sealed with polyethylene film and incubated at 30 °C for 7 days. The percentage of colony radial growth inhibition was calculated as described in Section 2.3.

Based on the initial screening of the individual VOCs, Ben and Dmo were selected for further study of their antifungal activity against *A. flavus* at different concentrations using the double Petri dish assay. Briefly, 60, 40, 20, 10, and 5 μL of the two selected compounds were pipetted separately on to each Petri dish. Assays were performed as described above, and the double Petri dishes were incubated at 30 °C for 7 days. The percentage of fungal growth inhibition was calculated as described in Section 2.3. For plates without *A. flavus* colonies, *A. flavus* spores were transferred from the inoculation site to fresh PDA plates. The plates were then incubated at 30 °C for another 7 days. Fungi that did not resume to grow were considered to have been killed. All experiments were conducted three times.

### 2.7. In Vivo Test of the Effect of Ben and Dmo on A. flavus Decay in Unhulled Rice

Ben, Dmo, and a mixture of the two compounds at a 1:1 volume ratio were tested for *A. flavus* control effect in unhulled rice using the Erlenmeyer flask incubation method described in Section 2.4. The test was slightly modified, and a 2 cm diameter filter paper was used instead of the NA medium. The tested VOC was added to the filter paper at a selected concentration based on the results of the test described in Section 2.6. Unhulled rice samples were used in this test, and the culture conditions and measurement of rice samples were the same as those described in Section 2.4.

### 2.8. Statistical Analysis

All data were analyzed using Microsoft Excel 2202 Build 16.0, and the mean and standard deviation were calculated. An analysis of variance for multiple comparisons was conducted using SAS V9.4 software. The differences among mean values were analyzed using Turkey’s multiple range tests and were considered significant at *p* < 0.05.

## 3. Results

### 3.1. Bacillus Strain Isolation and Screening of Strains Producing High Antifungal Active VOCs

**Figure 2:**
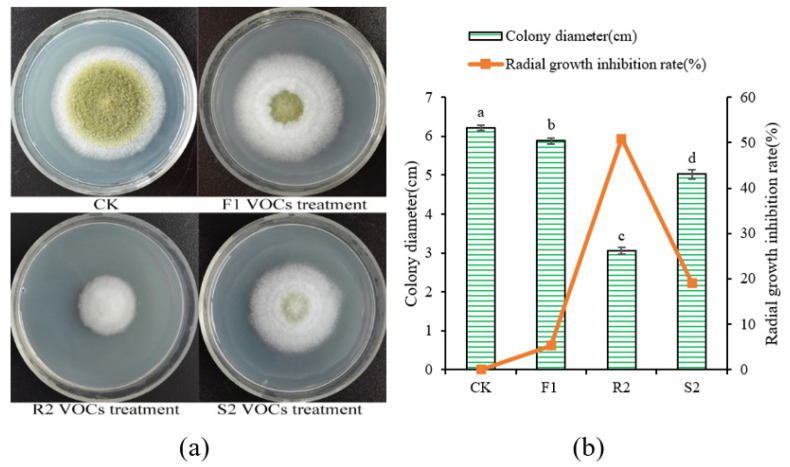
Effect of VOCs produced by isolated Bacillus strains on the radial growth of A. flavus. (**a**) *A. flavus* was treated by VOCs produced with different isolated strains and cultured on PDA plates at 30 °C for 5 days. (**b**) The colony diameter and radial growth inhibition rate of *A. flavus* treated by different VOCs produced by the isolated strains and cultured at 30 °C for 5 days. The mean values with different letters are significantly different (*p* < 0.05).

Three *Bacillus* strains, F1 from bean curd, R2 from unhulled rice, and S2 from sauerkraut, were isolated. The VOCs produced by these strains were tested for antifungal activity using a double Petri dish assay (Figure 2a). Among the three isolated strains, R2 produced VOCs that most potently inhibited the growth of *A. flavus* (Figure 2b). Therefore, this strain was selected for further research.

### 3.2. Physiological, Morphological, and Biochemical Properties of Bacillus Strain R2

**Figure 3:**
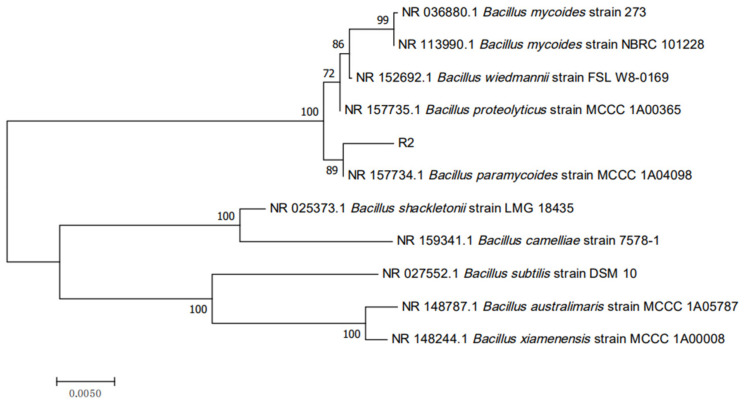
Phylogenetic tree of strain R2 based on 16S rDNA gene sequences. Bootstrap values expressed as percentages of 1000 replicates are shown at branch points.

Strain R2 was identified based on biochemical tests and 16S rDNA sequencing. Biochemical tests showed that the strain utilized sodium citrate and was positive for catalase, methyl red, starch hydrolysis, gelatin liquefaction, indole, glucose acid production, and VP tests, and was negative for phenylalanine deaminase, mannitol fermentation, and xylose acid production tests. These properties are in accordance with those of *Bacillus*. spp. PCR products of 1519 bp were amplified from the 16S rDNA of R2 genomic DNA, and NCBI BLAST results showed that this sequence was 99% similar to strain MCCC 1A04098 (NCBI accession number ON278015). In the NJ phylogenetic tree, the strain R2 clustered closely with the *B. paramycoides* strain MCCC 1A04098 (16S rRNA partial sequence length: 1509 bp) (Figure 3), with all neighboring strains belonging to the genus *Bacillus*, including *B. mycoides* strain 273 (1513 bp), *B. mycoides* strain NBRC 101228 (1477 bp), *B. wiedmannii* strain FSL W8-0169 (1540 bp), and *B. proteolyticus* strain MCCC 1A00365 (1509 bp). These results indicated that strain R2 belonged to the genus *Bacillus*.

### 3.3. In Vitro R2 VOC Inhibition of Conidial Germination of A. flavus

**Figure 4:**
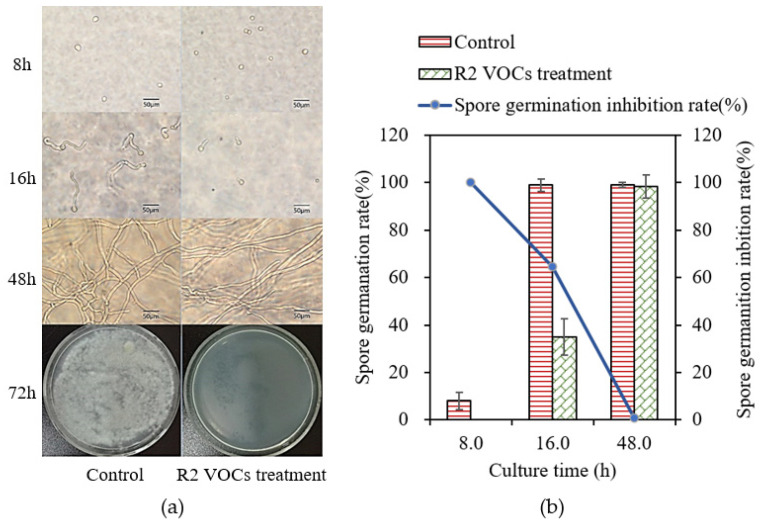
Effect of R2 VOCs on *A. flavus* conidia germination. An *A. flavus* conidia suspension was smeared on a PDA plate and cultured at 32 °C. (**a**) Spore germination of control and R2 VOC-treated *A. flavus* when cultured for 8, 16, 48, and 72 h respectively. Scale bar: 50 μm. (**b**) Spore germination rate and spore germination inhibition rate.

Conidial germination in the R2 VOC treatment and control plates was examined after culturing for 8, 16, 48, and 72 h in a double Petri dish. The results are shown in Figure 4a. When cultured for 8 h, the *A. flavus* spores in the control plates formed protrusions on the surface and began to germinate, whereas the spores in the treatment plates showed no signs of germination. When cultured for 16 h, almost all the spores in the control plates germinated, whereas the germination rate of the spores in the treatment plates was only 35%, as shown in Figure 4b, and the conidial germination inhibition rate was 64.6%. When cultured for 48 h, the hyphae of *A. flavus* in the control plates developed mycelia with many branches, whereas most spores in the treatment plates had germinated, but the mycelium did not form branches. When cultured for 72 h, white hyphae were visible to the naked eye on the control plates, whereas no hyphae were visible on the treatment plates. These results indicated that R2 VOCs inhibited both the germination of *A. flavus* spores and hyphal growth.

### 3.4. Effect of R2 VOCs on Rice Samples

**Figure 5:**
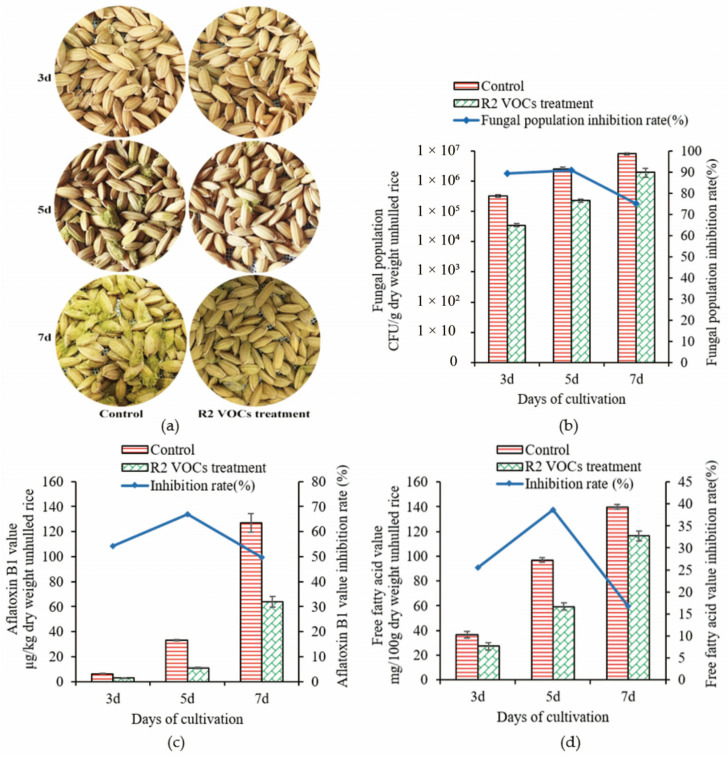
Effect of R2 VOCs on unhulled rice mildew. (**a**) Unhulled rice samples treated for 3, 5, and 7 days. (**b**) Inhibition effect of R2 VOCs on the fungal population in unhulled rice. (**c**) Effect of R2 VOCs on AFB_1_ levels in unhulled rice. (**d**) Effect of R2 VOCs on the FFA in unhulled rice. Data represent the means ± standard deviation from three biological replicates measurements.

Unhulled rice samples were inoculated with *A. flavus* conidia suspensions and stored at 32 °C for 3, 5, and 7 days. After culturing for 3 days, little molds on the rice were observed in both the control and treated samples. When cultured for 5 and 7 days, there were fewer *A. flavus* colonies in the treated samples than in the control (Figure 5a). Fungal populations in the rice samples of the treatment and control groups cultured for 3, 5, and 7 days are shown in Figure 5b. The inhibition rates of the fungal population in treated rice samples cultured for 3, 5, and 7 days were 89.4%, 90.8%, and 75.2%, respectively, compared with those in the control. The inhibition rate decreased when cultured for 7 days compared with that after 5 days, probably due to the reduction in VOC levels when R2 cells reached the decay phase and no more VOCs were produced.

AFB_1_ levels in the treated and control samples were determined using an ELISA kit (Figure 5c). When cultured for 3 days, the AFB_1_ levels in the treated and control samples were 2.86 and 6.26 μg/kg dry weight unhulled rice, respectively. With time, AFB_1_ levels increased in both the treated and control groups, yet the AFB_1_ levels in the treated samples were 67.1% and 49.8% lower than those in the control at 5 and 7 days, respectively.

With a series of biochemical changes in rice during storage, the fat was gradually decomposed to produce FFAs, resulting in an increase in the FFA levels, thus affecting rice quality. The FFA levels are an important indicator for determining the suitable storage time for rice [38]. FFA values in the treated samples were reduced by 26.2%, 38.7%, and 16.7% when the samples were cultured for 3, 5, and 7 days, respectively (Figure 5d). When cultured for 7 days, the reduction effect was weakened compared with that after 5 days, which was in agreement with the changing trends in the fungal population in the rice sample. This result indicates that the FFA values in the rice samples were closely related to rice mildew.

### 3.5. Composition of VOCs Produced by Strain R2

Eighteen compounds, including six alkanes, five alcohols, two esters, two cyano compounds, one aromatic aldehyde, and two other compounds, were detected through the HS-SPME-GC–MS analysis of strain R2 after growth in the NA medium (Table 1).

### 3.6. Inhibitory Effect of the Individual Compounds against A. flavus

Individual volatiles were pipetted at 80 μL/dish and the inhibitory effect against *A. flavus* was determined using double Petri dish tests. Notably, Ben was the most potent compound against *A. flavus*, as no hyphal growth was visible on the plate and the inhibition rate was 100%. Dmo was the strong active compound, and demonstrated an inhibition rate of 91.2%, whereas 2-methyl-2-oxazoline showed weak activity, with an inhibition rate of 20.6%. In contrast, 2,6-dimethyl-nonane and other compounds, such as n-nonadecane and n-pentadecane, showed no activity against *A. flavus*. Therefore, different concentrations of Ben and Dmo were selected for further experiments on antifungal activity.

### 3.7. Antifungal Effect of Different Ben and Dmo Levels against A. flavus In Vitro

**Figure 6:**
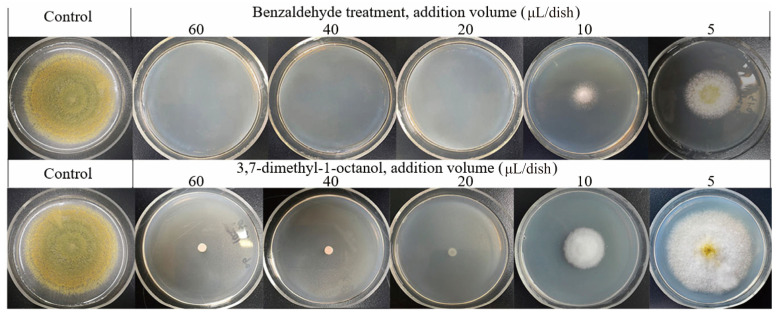
Effect of Ben and Dmo at different volumes on *A. flavus* growth in PDA plates incubated at 30 °C for 7 days.

Ben and Dmo were tested for their activity against *A. flavus* at different levels. Notably, no *A. flavus* growth was visible in the plates at a concentration of 60, 40, or 20 μL/dish of Ben, and the inhibition rate was 100% when cultured for 7 days (Figure 6). In the presence of 10 μL/dish of Ben, no *A. flavus* growth was observed during the first 4 days; however, the colony became visible on day 5 and the inhibition rate was 72.59% on day 7. The inhibitory effect was attenuated during cultivation at low concentrations, which might be due to the decrease in Ben concentration resulting from volatilization. After culturing for 7 days, *A. flavus* spores that could not form visible colonies on the treatment plates were transferred to fresh PDA medium and cultured at 30 °C for another 7 days. *A. flavus* conidia were completely killed by Ben at 60 and 40 μL/dish, whereas the 20 μL/dish of Ben completely inhibited *A. flavus* growth, but the treated spores resumed growth on fresh PDA plates.

Dmo could not inhibit *A. flavus* completely, even at the highest experimental concentrations. Only a very small amount of hyphal growth could be observed at the inoculation point at Dmo concentrations of 60, 40, and 20 μL/dish. The colony diameter did not increase during cultivation, and the inhibition rate was 91.2% at these concentrations. Furthermore, the inhibition rate was 66.7% and 14.9% at Dmo concentrations of 10 and 5 μL/dish, respectively, when cultured for 7 days.

### 3.8. Inhibition Effect of Ben and Dmo against A. flavus in Unhulled Rice

**Figure 7:**
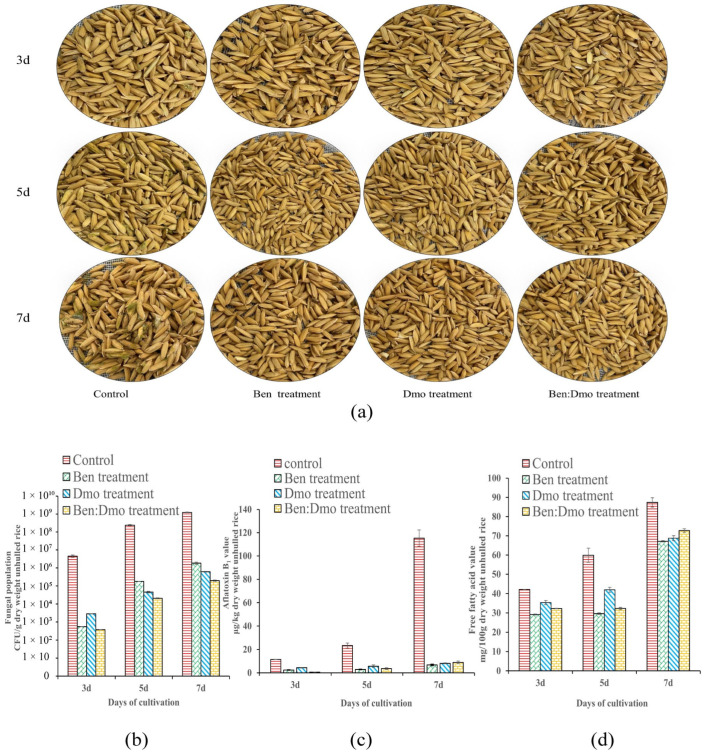
Effect of Ben and Dmo and their mixture in a 1:1 volume ratio on A. flavus decay in unhulled rice. The concentration of Ben and Dmo was 0.09 mg/mL and 0.072 mg/mL, respectively. In the mixture, the concentration of Ben and Dmo was 0.045 and 0.036 mg/mL, respectively. (**a**) Unhulled rice samples treated for 3, 5, and 7 days. (**b**) Fungal population in control and treated unhulled rice. (**c**) AFB_1_ values in control and treated unhulled rice. (**d**) FFA values in control and treated unhulled rice.

Based on the antifungal effect of Ben and Dmo at different concentrations in vitro, the two compounds were tested for the in vivo antifungal effect at 20 μL/dish, corresponding to a concentration of 0.09 mg/mL for Ben and 0.07 mg/mL for Dmo (Ben and Dmo density was 1.043 and 0.828 g/mL, respectively, and the diameter and height of the dish were 9 and 1.8 cm, respectively). Another mixture of the two compounds (Ben at 0.045 mg/mL and Dmo at 0.036 mg/mL) was tested for its antifungal effect (Figure 7a). The results showed that compared with the control, the fungal population and AFB_1_ and FFA levels in all treated samples decreased on days 3, 5, and 7 of culture. Among the three treatments, the Ben–Dmo-treated unhulled rice exhibited the lowest fungal population at 5 and 7 days (Figure 7b), AFB_1_ content was effectively controlled in all three treatments (Figure 7c), and the Ben-treated unhulled rice exhibited the lowest FFA levels at 3, 5, and 7 days (Figure 7d). 

## 4. Discussion

Traditional chemical methods used for fungal control during grain storage result in the development of drug resistance in pathogenic fungi, and their residues pose hazards to consumers. Therefore, alternative methods are needed to reduce the use of traditional chemical products. Antifungal VOCs are a promising strategy for controlling pathogenic fungi and related mycotoxins during grain storage. In recent years, *Bacillus* VOCs have been widely used for postharvest pathogen control [39,40,41]. Although the in vivo effects of *Bacillus* VOCs on fruits and vegetables such as cherry, citrus, loquats, and potato have been extensively studied [33,41,42,43], studies on *Bacillus* strains for controlling harmful fungi in stored rice grains remain limited. In the present study, we isolated strain R2 from unhulled rice and found that this strain produces highly active antifungal VOCs. Consequently, we sought to characterize the VOC_S_ produced by this strain and determine their control effect on *A. flavus* in vitro and unhulled rice.

Strain identification results showed that R2 was most closely related to *B. paramycoides*, which is a species isolated from the sediment of the South China Sea [44]. This species is effective against a variety of pathogenic fungi. The rhizobacterial strains *B. paramycoides* Vb3 and Vb6 are effective in controlling *Fusarium oxysporum* [45], whereas *B. paramycoides* R5 exhibits inhibitory action against the red rot pathogen *Colletotrichum falcatum* [46] and *B. paramycoides* JYZ-SD5 exhibits antagonistic activity against *Alternaria sp* and *Pestalotiopsis versicolor* [47]. However, the mechanisms underlying the antifungal activity of *B. paramycoides* have seldom been elucidated. In this study, the VOCs produced by strain R2 were identified and the main antifungal compounds among the VOCs were found out. These results provide new support for understanding the promising biocontrol effect of *B. paramycoides*. The HS-SPME-GC-MS results showed that the VOCs produced by R2 were mainly alkanes, alcohols, aldehydes, and esters. Among these, Ben was the most potent compound against *A. flavus,* with an inhibition rate of 100% at a concentration of 20 μL/dish when cultured at 30 °C for 7 days, and it killed *A. flavus* completely at 40 μL/dish. Ben has been reported to be a promising bioagent against pathogenic fungi [48,49], as it targets cellular antioxidant components of fungi [49] and shows strong inhibitory effects on these microorganisms, including *Monilinia fructicola* [48], *A. fumigatus*, *A. flavus*, *A. terreus*, and *Penicillium expansum* [49]. The HS-SPME-GC-MS results showed a relatively high Ben content in the R2 VOCs, demonstrating that Ben is a key antifungal active compound in the VOCs produced by R2. Dmo was another compound among the identified R2 VOCs individuals with an inhibition rate of 91.2% against *A. flavus* at a concentration of 20 μL/dish. To the best of our knowledge, this is the first report of Dmo’s antifungal activity. Some linear chain alkanes such as n-pentadecane and n-heptadecane have inhibitory effects on the mycelial growth of *Metarhizium anisopliae* and *Beauveria bassiana* [50]. Interestingly, in our study, both compounds showed no activity against *A. flavus* at test concentrations of 80 μL/dish (corresponding to 0.35 and 0.70 μL/mL). Even when the test concentrations were increased to 160 and 320 μL/dish, no inhibitory effects were observed.

The reported antimicrobial concentrations of Ben determined using different methods significantly differ among studies. A previous study reported the minimum inhibitory concentration (MIC) of Ben against *A. flavus* to be >35 mM (according to 3.71 mg/mL or 3.56 μL/mL) in assays performed using 96-well microtiter plates [51]. Ben (100 μL/plate) inhibited the growth of most *Clavibacter michiganensis* ssp. *sepedonicus* colonies in the I-plate system [52]. In the present study, *A. flavus* was completely inhibited by Ben at a 0.09 mg/mL headspace concentration (corresponding to 20 μL/plate) using a double Petri dish assay, which is much lower than previously reported values [53]. Another study investigated the MIC of heptanal against *A. flavus* via vapor fumigation and liquid contact culture. The MICs of heptanal using a liquid contact method and vapor fumigation were 0.8 and 0.085 μL/mL, respectively [54]. These results indicate that the antifungal effects of VOCs are affected by the method used. The antifungal volatiles showed more potent antifungal activity via vapor fumigation than liquid contact method.

Ben and Dmo showed excellent antifungal activity in this study, and their safety was verified by the Flavor Extract Manufacturers’ Association. These compounds provide options for researchers to explore environmentally friendly fumigants for grain storage. Compared with the traditional chemical antifungal agents, such as sodium diacetate [55] and propionic acid [56], Ben and Dmo do not need to be sprayed directly on the grain due to their high volatility, and they have the advantages of convenient use and low residues. Furthermore, as a traditional antifungal agent, propionic acid can control the fungal population in grains, but it has a poor control effect on FFA levels [57]. Our research shows that Ben and Dmo have control effects not only on the fungal population and AFB_1_ content, but also on FFA content in unhulled rice.

Although the present study has shed light on the potential antifungal properties of Ben and Dmo in unhulled rice, their optimal concentration for the control of harmful fungi in unhulled rice is still unknown and should be determined in the future. In addition, further investigation is needed to determine whether the antifungal activity of Ben and Dmo will be affected by different factors, such as environmental temperatures, humidity, and rice moisture content. In terms of safety, even though Ben and Dmo are a food grade synthetic flavoring allowed by the Flavor Extract Manufacturers’ Association, when they are used in unhulled rice, their potential impact on the environment and human health also requires a comprehensive safety assessment.

## 5. Conclusions

Our study presents experimental evidence demonstrating the efficacy of VOCs produced by *Bacillus* strain R2 in inhibiting *A. flavus* growth in vitro and mitigating its impact on unhulled rice. Specifically, among the R2-derived VOCs, Ben and Dmo exhibited robust antifungal activities against *A. flavus*, leading to reduced fungal populations and AFB_1_ and FFA levels. These findings suggest that Ben and Dmo could serve as promising alternatives to conventional chemical fumigants for controlling *A. flavus* contamination in stored grains. Moreover, further investigation is necessary to comprehensively evaluate the influence of Ben and Dmo on a wider range of toxin-producing fungi and their mycotoxin production in stored grains under diverse temperature and humidity conditions. Additionally, the potential impacts of residual compounds resulting from their volatilization on grain quality, as well as on human health and safety, warrant careful consideration in future studies.

## Figures and Tables

**Table 1:** VOCs produced by strain R2.

Compound	Chemical Formula	Retention Time (min)	Peak Area (%)
2,4-Dimethylheptane	C_9_H_20_	4.51	0.716
2,4-Dimethyl-1-heptene	C_9_H_18_	5.34	0.256
2,6-Dimethyl-nonane	C_11_H_24_	8.58	3.227
3,7-Dimethyl-1-octanol	C_10_H_22_O	10.12	0.211
Benzaldehyde	C_7_H_6_O	10.64	2.023
4-Carbonitrile-pyrazole	C_4_H_3_N_3_	10.98	0.485
3-Ethyl-3-methyl heptane	C_10_H_22_	12.71	0.306
n-Pentadecane	C_15_H_32_	13.1	0.584
n-Nonadecane	C_19_H_40_	13.63	1.027
2-Butyl-1-octanol	C_12_H_26_O	13.75	0.196
n-Hexadecane	C_16_H_34_	13.98	0.431
3,3-Dimethyl-1-butanol	C_6_H_14_O	14.25	0.535
2,3-Butanediol	C_4_H_10_O_2_	14.74	0.199
2-Methyl-2-oxazoline	C_4_H_7_NO	15.46	0.579
3-Butyn-1-ol	C_4_H_6_O	16.85	0.212
2-Methylglutaronitrile	C_6_H_8_N_2_	17.24	1.858
Tert-butyl nonaneperoxoate	C_13_H_26_O_3_	17.55	0.122
Ethyl propiolate	C_5_H_6_O_2_	20.15	0.476

## Data Availability

The original contributions presented in the study are included in the article, further inquiries can be directed to the corresponding author.
